# Vasculitis-Like Hemorrhagic Herpes Zoster and HIV Infections: An Intricate Association

**DOI:** 10.7759/cureus.50609

**Published:** 2023-12-15

**Authors:** Elena Codruța Cozma, Laura Mădălina Banciu, Cristina Soare, Mihnea-Alexandru Găman, Vlad Mihai Voiculescu

**Affiliations:** 1 Pathophysiology, University of Medicine and Pharmacy of Craiova, Craiova, ROU; 2 Dermatology, Elias Emergency University Hospital, Bucharest, ROU; 3 Hematology, Center of Hematology and Bone Marrow Transplantation, Fundeni Clinical Institute, Bucharest, ROU; 4 Hematology, Carol Davila University of Medicine and Pharmacy, Bucharest, ROU; 5 Dermatology, Carol Davila University of Medicine and Pharmacy, Bucharest, ROU

**Keywords:** bnt162b2 (pfizer-biontech), covid 19, cutaneous vasculitis, hiv, herpes zoster virus

## Abstract

Herein, we report the case of a 69-year-old patient who presented to our dermatology clinic for a skin eruption characterized by grouped hemorrhagic vesicles and erosions covered by hemorrhagic crusts on an erythematous background located on the lower right limb. The lesions were small, clustered, and variable in size (diameters between one and 10 mm) and located at the level of the L4-L5 dermatomes. The rash had started three to five days after the complete COVID-19 vaccination scheme with the BNT162b2 Pfizer BioNTech vaccine and had been accompanied by a flu-like syndrome. The histopathological examination established the diagnosis of leukocytoclastic vasculitis potentially in the context of a cytopathic zoster phenomenon. The atypical aspect of the zosterian eruption required additional laboratory work-up to identify possible causes of immunosuppression, i.e., screening for the presence of the human immunodeficiency virus (HIV) infection, solid cancers, as well as measurement of serum immunoglobulin concentrations, which revealed that the subject was HIV-positive. Antiviral treatment was started, with a favorable evolution of the lesions, and the patient was referred to an infectious diseases clinic for initiation of antiretroviral therapy (ART).

## Introduction

Human immunodeficiency virus (HIV) infection first appeared in 1981 in the United States through manifestations of opportunistic infections or neoplasms in young men who had sex with men or who used intravenous drugs [1]. Epidemiologically, HIV infection has so far been responsible for more than half a million deaths in both children and adults of both genders due to HIV-related causes according to the 2022 report of the World Health Organization regarding the global HIV epidemic [2]. However, despite the alarming increase in cases in the 1980s, with a peak in 1997, effective therapy and understanding of transmission methods have made it possible to reduce cases by up to 50% in the last years. The introduction of triple antiretroviral therapy (zidovudine + didanosine, zidovudine + zalcitabine, zidovudine + lamivudine) in 1996 displayed effectiveness in the long-term and reduced mortality and morbidity of the HIV infection. At that time, the HIV epidemic was severely affecting the healthcare system in the United States and African countries [3].

## Case presentation

The initial nonspecific clinical picture with an influenza-like syndrome, the long period of the viral set point that can last for years, as well as the subsequent polymorphic manifestations, make the diagnosis of HIV infection challenging. Skin manifestations are one of the most common complications of the disease, covering a wide range of manifestations, from bacterial and viral infections to malignancies and rashes in the context of drug toxicity. Of these, herpes zoster, caused by the reactivation of the varicella-zoster virus (VZV), can indicate the presence of an HIV infection. Shingles are 15 to 19 times more common in HIV-positive patients than in the general population and may present atypically in the setting of HIV infection, for example, verruciform cutaneous lesions with hyperkeratosis or necrosis of the integument [4,5].

We present the case of a 69-year-old male who presented to our dermatology clinic with a painful, pruritic rash on the right lower limb with a two-week onset. The medical history of the subject was relevant for the presence of a non-secretory pituitary adenoma diagnosed 20 years ago and treated with radiotherapy and transsphenoidal surgical excision. As a result of these interventions, the patient developed pituitary insufficiency on the gonadotropic line and was prescribed substitution treatment with testosterone with a favorable evolution.

**Figure 1:**
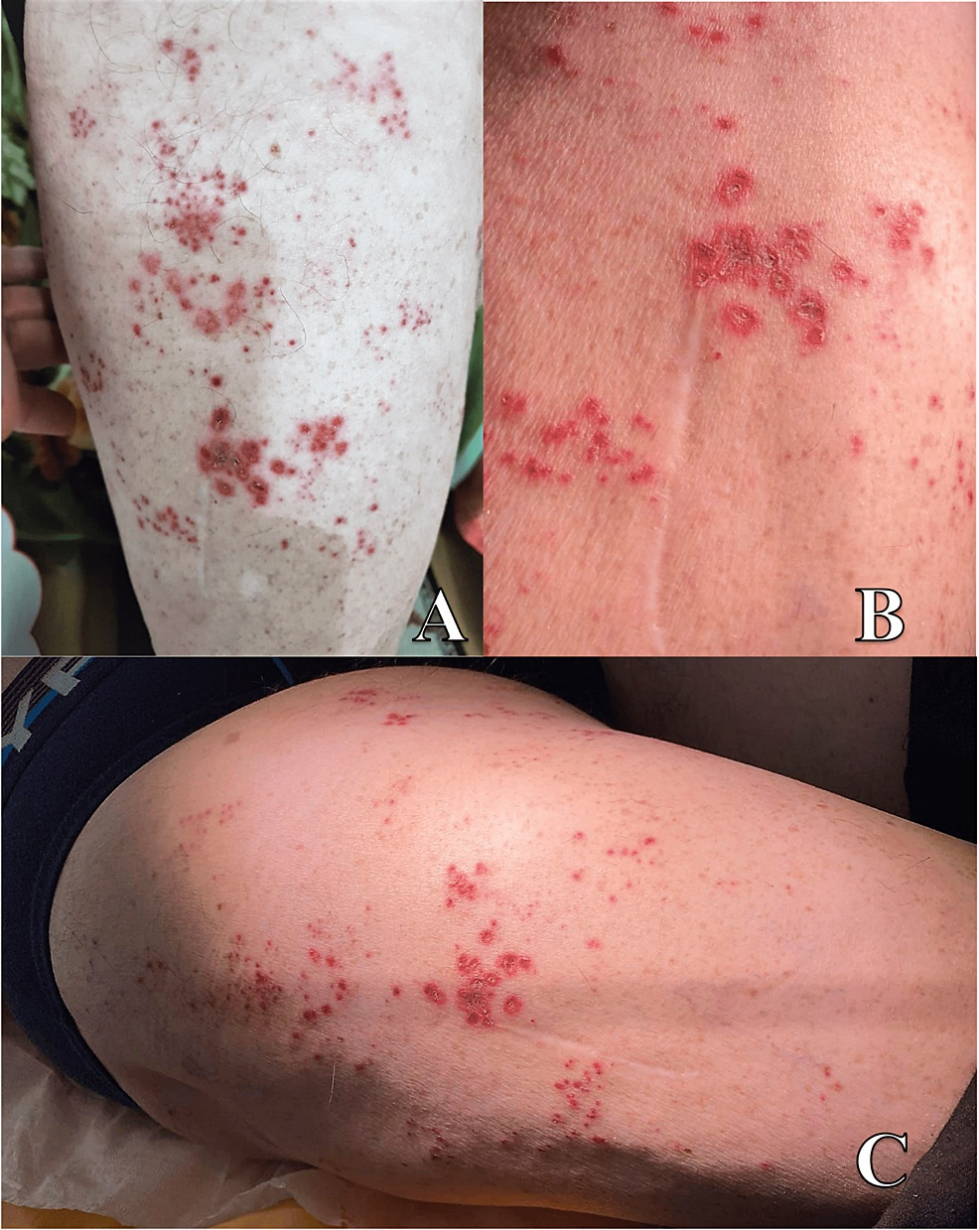
Clinical aspects of the lesions The clinical aspect of the lesion at the beginning (A) and at the moment of presentation in our clinic (B, C). Grouped, herpetiform lesions separated through apparently intact skin located mainly at the level of the L4 and L5 dermatomes of the right lower limb. The evolution toward erosions covered by hemorrhagic crusts of variable sizes (diameter of 1-10 mm) is observed.

The dermatological examination identified a rash consisting of small sanguinolent collections of hemorrhagic vesicles, erythematous macules, and erosions covered by hemorrhagic crusts, of variable sizes (diameters between one and 10 mm). The lesions were grouped, dermatomal, separated through apparently intact skin, and located mainly at the level of the L4 and L5 dermatomes of the right lower limb (Figure 1A-C).

Otherwise, the clinical examination was within normal limits. The onset of the rash occurred within three to five days after complete COVID-19 immunization with the BNT162b2 Pfizer-BioNTech vaccine. The patient also reported the presence of a flu-like syndrome (myalgia, distress) before the onset of the rash, as well as the presence of local pain and burning in the affected limb. The patient had no history of SARS-CoV-2 infection. Laboratory findings before the presentation in our clinic, performed in another medical facility, revealed elevated markers of inflammation, presence of pancytopenia, elevated D-Dimer concentrations, and decreased free S protein levels (Table 1).

**Table 1:** Main laboratory findings before the presentation in the dermatology clinic. mmc = millimeter cube.

Measured parameter	Normal lab values	Measured values
Erythrocyte sedimentation rate	<15 mm/1 h	31 mm/1 h
Fibrinogen	170–380 mg/dL	431 mg/dL
C-reactive protein	<0.5 mg/dL	0.56 mg/dL
D-dimers	<0.55 mg/L	4.12 mg/L
Free protein S	67.5–103%	63.5%
Leukocytes	3910–10,900/mmc	2480/mmc
Neutrophils	1800–6960/mmc	1370/mmc
Eosinophils	30–590/mmc	20/mmc
Thrombocytes	150,000–450,000/mmc	138,000/mmc
Lymphocytes	1260–3350/mmc	890/mmc

**Figure 2:**
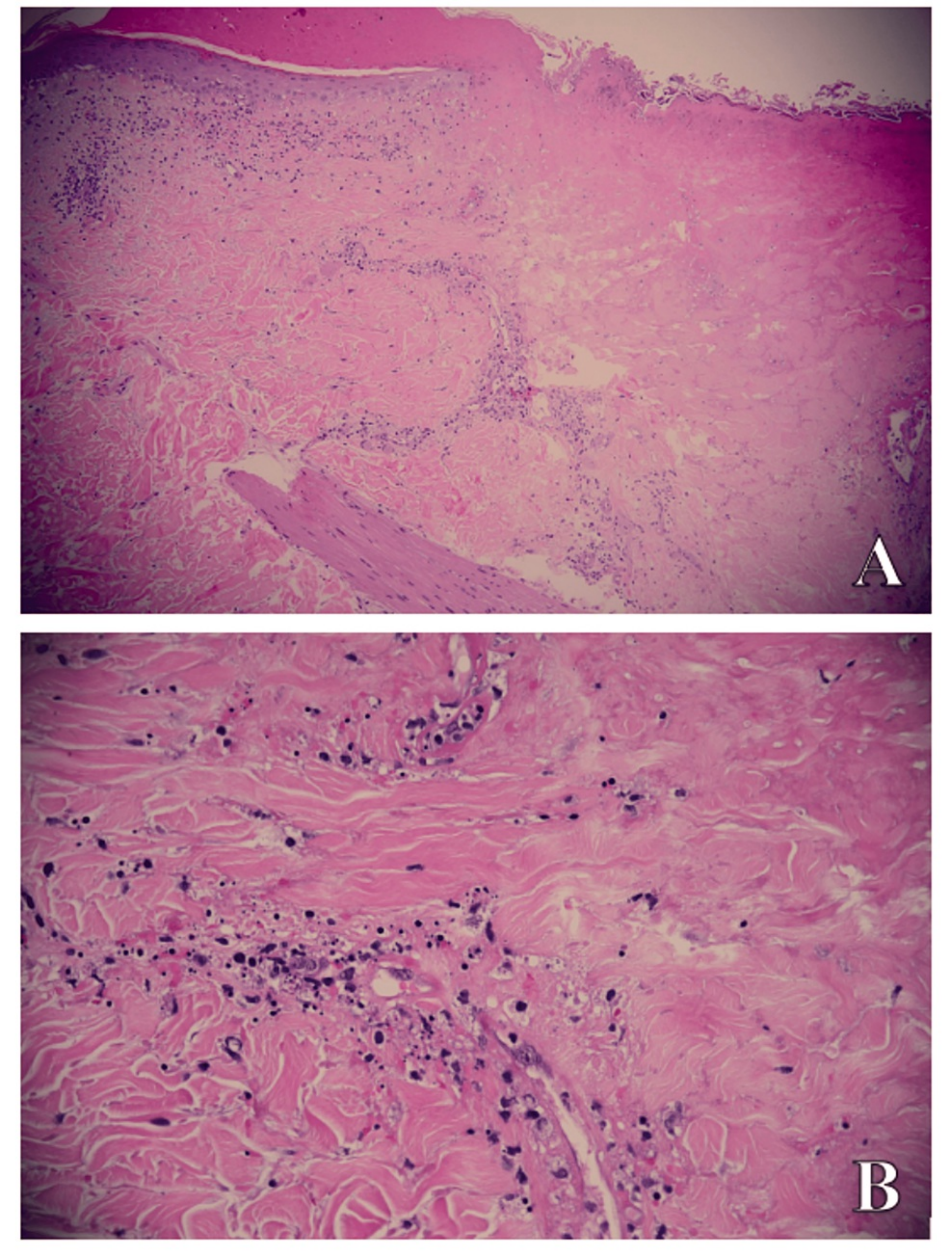
Histological aspects of a 6-mm punch biopsy specimen at the level of the lesions. (A) x5, (B) x10. Ulceratated lesion covered by fibrin-leukocyte and necrotic crust. Frequent neutrophilic and lymphocytic perivascular infiltrates with nuclear residues, extravasation of erythrocytes, and fibrinoid necrosis. Optical microscopy examination; hematoxylin-eosin stain.

Due to the clinical aspect and the unilateral distribution, which met both the criteria for vasculitis and shingles, we decided to perform a punch biopsy. The pathology report established the diagnosis of leukocytoclastic vasculitis as a potentially isotopic phenomenon to the development of shingles (Figure 2A,B).

Given the low frequency of this disorder, its occurrence in immunosuppressed patients or subjects suffering from blood cancers, as well as the laboratory data reported above, the patient was screened for the presence of HIV and tested positive for the infection. The subject denied the use of toxic substances with intravenous administration, as well as at-risk sexual behaviors in the past. The diagnosis of vasculitic shingles was established and was considered a revealing manifestation of latent HIV infection. Treatment with oral acyclovir (4 g/day for 7 days) was started with a favorable evolution of the lesions. The patient was referred to an infectious disease clinic for disease staging and initiation of specific antiretroviral treatment.

## Discussion

Vasculitic manifestations of shingles are rare and often associated with a severe prognosis. They generally affect the cerebral vessels and can cause damage to large and small vessels [6]. Shingles can affect both the central nervous system, causing encephalitis, and the peripheral nervous system, leading to post-herpetic neuralgia, segmental paresis, and Guillain-Barré or Hunt syndrome, respectively [7,8]. Leukocytoclastic vasculitis is a small vessel vasculitis responsible for approximately 37% of vasculitic-type phenomena due to VZV infection. However, manifestations of this type are extremely rare, especially distributed at the level of the limbs. Only a few cases of shingles-related vasculitic eruptions have been reported in the literature [9].

Differential diagnosis of our patient's lesions was difficult. The lesions could have been interpreted as leukocytoclastic vasculitis in the context of VZV infection (either post-vaccination or in the context of HIV infection) or as hemorrhagic shingles in the context of HIV-induced immunosuppression.

SARS-CoV-2 and VZV association

The SARS-CoV-2 virus has been the focus of medical attention for the past three years, with both the infection and the vaccine being associated with multiple complications or side effects that can present as skin manifestations. Thus, considering the anamnesis, the presence of the flu-like syndrome, and the short duration of onset after the second dose of the BNT162b2 Pfizer BioNTech vaccine, the first diagnosis considered was that of a post-vaccination reaction. There are several reported cases of zoster eruptions post-SARS-CoV-2 immunization. In these situations, the rash occurred more frequently in the first two weeks following the first dose, a phenomenon explained by a progressive post-vaccine lymphopenia, with a decrease in CD4+ and CD8+ lymphocytes responsible for cellular defenses that prevent the reactivation of the zoster virus from the dorsal ganglia roots [10]. Reactivations after the second dose are, however, less common, with most cases reported affecting the cephalic extremity [11,12]. However, our patient presented with moderate lymphopenia in the context of the HIV infection, which could explain a shingles reactivation following immunization on the background of pre-existing immunosuppression. This diagnosis does not change the therapeutic management of the current eruption.

Association of leukocytoclastic vasculitis and shingles

Another considered diagnosis was that of leukocytoclastic vasculitis occurring synchronously with shingles, affecting the same dermatomes, as a revealing phenomenon of HIV infection. Several pathophysiological mechanisms have been proposed to explain the development of this manifestation. Viral infections can be responsible for the occurrence of vasculitis by altering endothelial cells. In cases of synchronous occurrence, the most plausible mechanism is considered to be the invasion of blood vessels employing nerve fibers that run from the dorsal root to the vessel and along which the viral material is transported [13]. The distribution of the lesions and the histopathological aspect supported this diagnosis; however, due to the rarity of this manifestation, as well as the patient's inability to chronologically describe the evolution of the lesions, the post-shingles vascular cytopathic phenomenon became a more likely diagnosis [14].

Severe immunodepression and VZV association

The last diagnosis considered was that of hemorrhagic shingles as a revealing phenomenon of HIV infection that occurred in the context of immunosuppression and coagulation disorders caused by both thrombocytopenia and changes in S protein levels that the patient exhibited [15]. Hemorrhagic shingles is a rare condition, most often associated with the administration of antiplatelet or anticoagulant drugs, conditions that cause thrombocytopenia, or even with the SARS-CoV-2 infection [16,17]. No cases of hemorrhagic shingles associated with SARS-CoV-2 vaccines have yet been reported. Although affecting only the skin in this case, hemorrhagic shingles remains a life-threatening condition and can be complicated in a large number of cases by hemorrhagic encephalitis or mesenteric infarction, thus requiring early antiviral treatment and close follow-up [18,19]. To our knowledge, there has been another report of VZV reactivation in an HIV-positive individual after the second dose of the SARS-CoV-2 Pfizer vaccine; however, the patient was already in the acquired immune deficiency syndrome (AIDS) stage of the HIV infection, and thus not discovered with HIV primoinfection as in our case [20].

## Conclusions

In our case, shingles was a harbinger of HIV infection. The patient was diagnosed with secondary leukocytoclastic vasculitis, which manifested as a vasculitic-like rash and hemorrhagic vesicles. In the absence of the patient's SARS-CoV-2 immunization, which could have exacerbated the lymphopenia and accelerated viral reactivation, we cannot infer the length of time for which the HIV infection could have remained dormant, thus delaying the administration of antiretroviral therapy. Thus, even if shingles is a common condition in people over 60 years, atypical manifestations should warrant investigation for underlying conditions.
